# Perceived influences on reducing prolonged sitting in police staff: a qualitative investigation using the Theoretical Domains Framework and COM-B model

**DOI:** 10.1186/s12889-021-12019-6

**Published:** 2021-11-19

**Authors:** Marsha L. Brierley, Lindsey R. Smith, Daniel P. Bailey, Sofie A. Every, Taylor A. Staines, Angel M. Chater

**Affiliations:** 1 Centre for Health, Wellbeing and Behaviour Change, Institute for Sport and Physical Activity Research, University of Bedfordshire, Polhill Avenue, Bedford, MK41 9EA UK; 2 Division of Sport, Health and Exercise Sciences, Department of Life Sciences, Brunel University London, Uxbridge, UB8 3PH UK; 3 Sedentary Behaviour, Health and Disease Research Group, Brunel University London, Kingston Lane, Uxbridge, UB8 3PH UK; 4 Centre for Behavioural Medicine, University College London, Tavistock Square, London, WC1H 9JP UK

**Keywords:** Sedentary behaviour, Intervention, Office workers, Barriers, COM-B, Qualitative, Police

## Abstract

**Background:**

Workplace interventions have shown promise for reducing sitting in office workers. Police office staff remain an understudied population group that work within a disciplined organisation with distinctive work tasks around public safety, potentially affecting their capability, opportunity, and motivation to change sitting behaviour. This study aimed to assess the perceived influences on reducing workplace sitting in non-operational, desk-based police staff in order to derive theoretical determinants for behaviour change.

**Methods:**

Ten police staff from a single police force in Bedfordshire, England [eight female; 39.5 ± 11.5 years] took part in face-to-face semi-structured interviews lasting 46 ± 11 min on average. Thematic analysis identified key themes which were then mapped onto the Theoretical Domains Framework (TDF) and linked to the Capability, Opportunity, Motivation-Behaviour (COM-B) model.

**Results:**

Seven themes were identified: *‘Work tasks are seated’, ‘Social norm is to sit’, ‘Belief in ability to regulate behaviour’, ‘Knowledge of health risks’, ‘Organisational support’, ‘Impact on productivity’, and ‘Perceived autonomy for sitting reduction’*.

**Conclusions:**

Awareness of behaviour and health impacts (Capability), social and physical support to sit less (Opportunity), and habit formation techniques (Motivation) are recommended considerations in sitting reduction workplace interventions for police staff.

**Supplementary Information:**

The online version contains supplementary material available at 10.1186/s12889-021-12019-6.

## Background

In order to reduce the risk of developing chronic disease, experts recommend that full time, desk-based workers progressively replace half of their working day with standing and light intensity activity (e.g., light walking), as well as engage in regular breaks from static seated or standing work [1]. High amounts of sedentary behaviour are associated with poor health outcomes such as increased risk of cardiovascular disease, obesity, type 2 diabetes, some cancers, and premature mortality [2–6]. Office work is a known environment for prolonged sedentary behaviour (sitting) and has become a target for behaviour change interventions [7]. A study involving 5527 British police force employees reported that 30% had mainly office-based duties [8]. While barriers to sitting reduction for office workers in retail, call centres, health, and information technology have been previously documented [9, 10], no information exists as to the specific needs of non-operational, desk-based police staff. The disciplined nature of the organisation and distinctive work tasks around public safety may lead to unique influences on behaviour for police staff.

Multiple influences act (and interact) on sitting behaviour including environmental, social, political, and individual-level factors [11]. The office environment can limit movement during work hours [12] and a culture of expectation that equates work completion with sitting at one’s desk has been shown to influence employee behaviour [13]. Workplace health and safety policies also widely encourage sitting behaviour [14], while individual-level factors such as age and mood have also been found to influence sitting behaviour in working age adults [15]. For the greatest chance of success, interventions need to be tailored to the target population, context, and setting by identifying the key influences of sitting behaviour in specific occupational groups [16, 17].

The Behaviour Change Wheel (BCW) is a multi-layer framework for intervention development that incorporates the Capability, Opportunity, Motivation-Behaviour (COM-B) model for understanding behaviour change [18, 19]. The COM-B model can be used to understand one’s physical and psychological capability for reducing occupational sedentary behaviour, one’s social and physical opportunity for doing so, and one’s reflective (conscious) and automatic (non-conscious) motivations towards the behaviour. The benefit of this approach is that the BCW builds on prior paradigms that consider multiple levels of influence (i.e., the social ecological model) by allowing for mechanistic modelling of how and why behaviour is likely to be influenced [18, 19]. By considering a comprehensive range of influences on behaviour, the BCW extends the use of individual psychological approaches, such as social-cognitive theory, which do not necessarily consider additional levels of influence on behaviour, such as environment and policy. The resulting ‘COM-B Behavioural Diagnosis’ can also be linked to psychological theory represented by the Theoretical Domains Framework (TDF) [20, 21]. The TDF is an amalgamation of 33 psychological theories of behaviour change and is useful in expanding the COM-B elements to gain a more detailed understanding of what might influence sitting behaviour [20]. Identifying the COM-B and TDF domains is useful to intervention designers because such a behavioural diagnosis enables tailoring intervention strategies and selecting appropriate behaviour change techniques which can be evaluated for efficacy [22]. The COM-B model, in conjunction with the TDF has been used successfully in the past for the development of sedentary behaviour interventions [22–24], but it has yet to be applied in the context of police staff.

The aim of the current study was to assess the perceived influences on reducing and breaking up prolonged sitting at work in police staff. Identifying stakeholders’ challenges and opportunities using qualitative research methods allows for intervention tailoring to the unique needs of the target population. Identified themes can be mapped onto the COM-B and TDF, thus providing the evidence base for transparent decision-making during the intervention development process [7, 22]. Elucidating the determinants of behaviour for this occupational subgroup will provide a clear evidence base from which future interventions may be informed.

## Methods

### Design and participants

This qualitative study was part of a larger project [the Police Health and Wellbeing (*PHeW*) project] conducted with a regional police force in the United Kingdom. A convenience sample of police office staff was recruited at a single police force in Bedfordshire between October 2018 and April 2019. Invitation emails were sent by a researcher involved in the wider project to all participants who had recently taken part in a *PHeW* health and fitness assessment. Interested individuals could click on a link in the email that took them to a secure website (Qualtrics Inc., Seattle, WA, USA) where the participant information sheet was provided. Staff were defined as police employees with non-operational, desk-based job roles as identified by the organisation’s human resources department. To be eligible, participants were required to be 18 years or over old, work more than 0.6 full-time equivalent hours, spend the majority of the workday seated according to self-report, not currently pregnant, able to walk, not taken part in a sedentary workplace intervention before, and not have had personal access to a height-adjustable workstation. Participants provided informed consent on the secure website, after which they were contacted by the lead researcher (MLB) for interview. Informed consent for the use of anonymous quotations was obtained from participants prior to interviews. Ethical approval was granted from the University of Bedfordshire Institute for Sport and Physical Activity Research Ethics Committee (approval no. 2018ISPAR014) and was performed in accordance with the Declaration of Helsinki for research involving human participants. There was no relationship between the potential participants and lead researcher prior to the study. It was disclosed during interviews that the researcher was designing an intervention for police staff. COnsolidated Criteria for REporting Qualitative studies (COREQ) guidelines were followed [25] (see Additional file 1, which shows the completed COREQ checklist).

### Materials

#### Demographic survey

All participants completed a 12-item demographic survey including questions on age, gender, education, and physical activity behaviour [26]; marital status; departmental job role; time in service; hours worked per week; perceived health; cardiometabolic medical conditions (cholesterol, diabetes, high blood pressure); smoking status; and alcohol consumption [27].

#### Interview schedule

A semi-structured interview schedule was used based on prior work [7, 9, 28] and iteratively developed [29] to explore participants’ perceived influences (e.g., barriers and facilitators) for reducing sitting at work (see Additional file 2, which shows the interview schedule). Topics included questions around job role and sedentary time; prior knowledge of sedentary behaviour health risks and benefits to workplace sitting time reduction; barriers and facilitators (including prior knowledge of strategies for reducing sedentary time); impacts on productivity and health; and finally, perceptions of interventions and behavioural monitoring.

### Procedure

Semi-structured interviews were conducted face-to-face at two sites (police headquarters and a large satellite office) during work hours (as approved by management). Participants represented a range of departments and included non-managers as well as managers, though sampling was not deliberate. Interviews were recorded using a Tascam DR-05 dictaphone (TEAC Corporation, Montebello, CA, USA) and transcribed verbatim using Express Scribe software (NCH Software, Inc., Canberra, Australia) and an Infinity IN-USB-2 foot pedal (AltoEdge Pty Ltd., Colorado, USA). Transcripts were de-identified and anonymised prior to analysis. After data immersion and regular discussions among the research team, saturation was agreed to have been met after 10 interviews, with no new themes being identified [30]. Interviews lasted an average of 46 ± 11 min.

### Analysis

Demographic statistics are reported as mean ± SD or percentages/frequencies. Interview data was thematically analysed [31] using Nvivo 11.4.3 (QSR International Pty Ltd., Melbourne, Australia). Researchers followed an inductive, iterative process whereby transcripts were first coded by MLB line by line, then sub-themes were identified, and finally, sub-themes were grouped together under key themes. Final themes were discussed and adapted by MLB and AMC and then confirmed with the wider research team [32]. Twenty percent of transcripts (*n* = 2) were blind-coded (by AMC) using a final themes codebook, with inter-rater reliability reported as very good (91.7%). All key themes were deductively mapped to COM-B, and all subthemes linked to TDF categories, using a framework matrix [7, 33]. In this way, coded data was summarised along with illustrative quotes, allowing for exploration of higher order themes while retaining the original context of the interviews [34].

## Results

### Participants

Ten police staff volunteered and gave consent to be interviewed for this study (eight female; 39.5 ± 11.5 years). Participant characteristics are shown in Table 1. Participants represented a variety of different departments across the Force including finance, corporate, and management (*n* = 4); Force control room and victim services (*n* = 2); intelligence (*n* = 2); and analytical services (*n* = 2). Seventy percent were married/cohabiting/had civil status (*n* = 7). Twenty percent reported having high cholesterol (*n* = 2), with no other cardiometabolic conditions reported.

**Table 1:** Demographic characteristics of interviewed police staff (*n* = 10)

Characteristic	Mean ± SD or ***n*** (%)
Sex (female)	8 (80%)
Age (years)	39.5 ± 11.5
Years in service	10.4 ± 8.2
Hours worked per week	37.3 ± 2.9
**Managerial duties**	
Manage others	6 (60%)
Do not manage others	4 (40%)
**Rank**	
Non-ranked	8 (80%)
Police Constable/Sergeant	2 (20%)
**Highest Education Level**	
A-level, high school, or equivalent	3 (30%)
University	6 (60%)
Postgraduate qualifications	1 (10%)
**Physical activity habits**	
Never exercise	2 (20%)
Less than 150 min/week	4 (40%)
Equal to 150 min/week	2 (20%)
More than 150 min/week	2 (20%)
**Self-rated health status**	
Excellent	0 (0%)
Very good	2 (20%)
Good	5 (50%)
Fair	3 (30%)
**Smoking status**	
Ex-smoker	1 (10%)
Current smoker	3 (30%)
Never smoked	6 (60%)
**Typical alcohol consumption**	
Never drink	2 (20%)
14 units or less per week	6 (60%)
More than 14 units per week	2 (20%)

### Themes

**Fig. 1:**
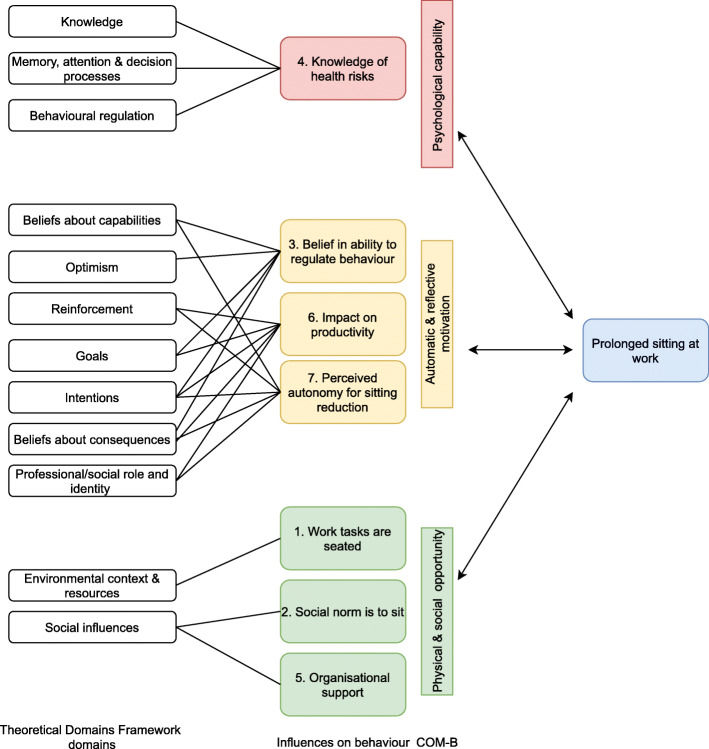
Key themes around influences on reducing workplace sitting time in police staff mapped to the Theoretical Domains Framework (TDF) and linked to the Capability, Opportunity, Motivation-Behaviour (COM-B) model (figure adapted from [7])

Seven themes were identified as influencing police staff’s sitting behaviour (i.e. breaking up and reducing prolonged sitting) at work: 1)*‘Work tasks are seated’,* 2)*‘Social norm is to sit’,* 3) *‘Belief in ability to regulate behaviour’,* 4) *‘Knowledge of health risks’,* 5) *‘Organisational support’,* 6) *‘Impact on productivity’,* and 7) *‘Perceived autonomy for sitting reduction’*. Fig. 1 shows the link between all key themes, TDF categories, and COM-B. Inductive themes are presented below with COM-B in brackets [] with participant quotes (identified by pseudonym, sex, and manager status).

#### Work tasks are seated [physical opportunity]

Police staff unanimously described performing work tasks that tied them to the desk.

*“The minority roles in the police are putting on a uniform and rushing around in blue lights. That’s what I used to do, but that’s the minority. The rest is behind-the-scenes places like what you can see here in offices, and that’s sat down doing work.” (P5, male, non-manager)*

*“Obviously it needs to be done on the computers. So that keeps you at your desk.” (P7, female, manager)*

Participants described typical office set ups, though an atypical instance of changes to a shared space was remarked on:

*“…no, like, standing desk options or anything like that.” (P1, female, non-manager)*

*“The whole thing [forensics room with standing working options] is virtually brand new. It’s been in the last year or two it’s been built.” (P5, male, non-manager)*

Meeting rooms were not generally set up for standing:

*“Sometimes you can be in [meeting] rooms and they’re rammed. So if you were standing you might be blocking someone else’s view or like it’s difficult to get out or it’s not physically possible.” (P8, female, manager)*

The close proximity of toilets, meeting rooms, and kitchen facilities was sometimes positively perceived as an excuse for a short break, and sometimes perceived as a lost opportunity for incorporating more movement during the workday.

*“The toilets are not far, but you do have to do a bit of a walk to get to them. So that’s good in a way because it just gives you that little break sometimes that you need.” (P1, female, non-manager)*

*“In our office we’ve got a kettle in the room, so we don’t tend to have tea breaks or anything. We’ll just get up and make a drink which, again, it’s not far from my desk so I do get up but, like, from here to you ((gesturing across the booth)) ((laughs)), to the kettle, make a cup of tea, and then sit back down again.” (P8, female, manager)*

Necessary security clearance led to micro breaks away from the desk in order to greet visitors, however, access restrictions were perceived to contribute to reduced movement overall.

*“‘Cos our office is a secure office, every time someone rings or knocks on the door, because I’m closest, I have to get up and get it. Which does help with the standing ‘cos some days - other than going to the toilet - that’s probably the only time I get up ((laughs)).” (P8, female, manager)*

Participants also talked about digital security clearance and sluggish software that kept them waiting, and therefore sitting, for longer.

*“This new computer system… that they’ve brought in, it has a massive impact on how we do our day-to-day business because it’s made it more drawn out.” (P10, male, manager)*

However, participants were wary of the potential cost implications for the Force of equipment like sit-stand desks.

*“We’re trying to save money so I couldn’t see that being an option at this moment in time.” (P7, female, manager)*

### Social norm is to sit [social opportunity]

Office culture was perceived as synonymous with sitting due to social norms, including sitting during lunch breaks for both managers and non-managers.

*“I have people under me, and they see me eating my lunch at my desk. So yeah, I think there needs to be a bit of a cultural shift on that one.” (P2, female, manager)*

*“Like, the managers especially, they’re predominately just sitting most of the day. […] I think, maybe, just the work they do? They have a lot of work to do so maybe they’re just trying to crack on with it.” (P3, female, non-manager)*

Participants who attended meetings reported that sometimes there had been good intentions in the past to incorporate standing or breaks.

*“Our initial [supervisor] who’s now left, and he was quite rigid in the way he wanted things. So he wanted it to be like a standing fifteen minute daily meeting. And since he’s left that has now reduced to twice a week, and a handful of people will stand, but most people just sit, ‘cos sometimes it can go on for a while. If you are standing, you’d eventually get a chair and sit down.” (P3, female, non-manager)*

Participants talked about the social awkwardness of standing in meetings and feeling the need for someone in charge to give permission to stand.

*“…you’d feel a little bit self-conscious, just kind of standing. Like drawing attention to yourself.” (P8, female, manager)*

*“No, not really [can we take breaks]. Unless they say, ‘Oh, we’ll take a leg stretch,’ which is a quick trip to the ladies and back again.” (P2, female, manager)*

*“Maybe the Chair [of the meeting]? Or someone like that, that’s, you know...” (P8, female, manager)*

#### Belief in ability to regulate behaviour [reflective and automatic motivation]

Participants often expressed reasons to change their behaviour and belief in their capabilities and consequences.

*“I just try and, you know, remind myself to get up. And I think, fortunately, I do sort of tend to get quite thirsty, so I do tend to naturally want to drink at least probably once an hour […]. I'm getting up and, you know, even if it’s just walking out to the kitchen to get a coffee or a water.” (P7, female, manager)*

*“I think in our office, I think we do understand. And I do stand up and walk over to the window, have a look out and then put the kettle on and… But it’s only a minute, two minutes. It’s not for any great amount of time and we do have room in our office to walk up and down a little bit, which we do quite frequently.” (P2, female, manager)*

However, not all employees felt the same way about changing their sitting behaviour.

*“I think I tend to get lazy […]. I just sometimes feel like ‘Oh, like, what’s the point?’ like ‘I might as well stay sitting here.’ My colleagues would be like, ‘Oh, you know, do you want to come down?’ And I’d be like, ‘Can’t be bothered’. So yeah, just sometimes doesn’t feel like there’s any point.” (P3, female, non-manager)*

Ambivalence about changing sitting behaviour at work was often described as being due to ingrained habits.

*“I think it would just take a bit of time to get into a new routine, I suppose. ‘Cos obviously, when you’ve been doing something for so long, you just have to break that habit, don’t you?” (P3, female, non-manager)*

A regular routine to reduce sitting, perhaps even a regular exercise class offered on-site, was cited as a potential way to establish reasonable work expectations.

*“It’s easier because it becomes part of your routine then. And it’s easier to fit it in because everyone knows, ‘Oh, where’s [participant’s name]?’ ‘Oh, she’s doing her Pilates. She’ll be back in an hour or whatever.’ It would just be easier.” (P9, female, manager)*

#### Knowledge of health risks [psychological capability]

The overall knowledge of health risks was largely limited to long term musculoskeletal issues (e.g., overuse injuries, back pain). Few participants mentioned the impacts of prolonged sitting on cardiometabolic health, and, when prompted, could not recall much information apart from weight gain.

*“Anyone who comes straight outta university now, straight into an office job, they come into our office, they’re gonna be sat. And they’re gonna have the complications with the skeletal earlier than I have done.” (P2, female, manager)*

*“If you’re sitting for long periods of time, you’re not moving. You’re not using any energy, or producing… Like your metabolism’s not working and stuff like that. So, I don’t know if it does?” (P6, female, non-manager)*

Though participants had knowledge of the potential benefits for wellbeing and work performance (e.g., concentration and energy levels), they often described having trouble remembering to get up.

*“Sometimes it, the time, just honestly, the time just goes, and you don’t know that you’ve been sat there for a whole hour and a half.” (P6, female, non-manager)*

*“I have to sort of try and remember to get up every hour or so to walk around. That’s what one thing I'm guilty of. Sometimes I sit here without moving for a long time.” (P7, female, manager)*

#### Organisational support [physical and social opportunity]

Participants generally felt positive about the organisational support available to employees who, for health reasons, require changes to their way of working (e.g., ergonomic adaptations for desk working due to an injury on the job or back pain).

*“I’ve seen one person on our corridor who has one [a sit-stand desk], but I think he was involved in some sort of on-duty accident. So I s'pose the Force feels that they need to do that.” (P2, female, manager)*

However, it was perceived that support was provided reactively, on a case-by-case basis, and that signposting could be improved.

*“Anyone can request a workstation assessment. […] To be honest, the [person] that used to do it has just left, so I don’t really know who it is. So maybe it would help if we knew who the people were, ‘cos I’d have to go searching for someone at the moment, I think. But there are people around.” (P2, female, manager)*

It was acknowledged that the Force provided information about how to reduce prolonged sitting, but participants felt a lack of organisational and occupational support to carry out the advice. Organisation-led initiatives were a suggested solution.

*“It says [reading a nearby A4 wall poster], ‘vary your activity to reduce fatigue, get a drink, talk on the phone with a colleague, stretch, and take a short walk for a few minutes every hour.’ See, that’s there. But who does it?” (P2, female, manager)*

*“I think if you did it like as a whole group… like as a group… to make everyone else aware of it, you know? I'm sure that it would help everybody.” (P6, female, non-manager)*

#### Impact on productivity [reflective and automatic motivation]

Participants expressed productivity concerns when it came to adding objects like seat cycles or treadmill desks because of fears that the physical effort might be distracting for work completion.

*“Maybe it would be hard to work and think ‘Keep cycling’ kind-of-thing?” (P1, female, non-manager)*

Sit-stand desks were perceived favourably as one could continue working but in a different (static) posture.

*“I feel like it [a sit-stand desk] could maybe boost my productivity because it’s a change in like the way I’m working, if that makes sense? So, it’s not like a change in the environment, but it is… stimulate me a bit more so […] I can’t really say but I think it might have a positive impact.” (P3, female, non-manager)*

Regularly breaking up sitting time during the day was perceived as having positive benefits.

*“I think it probably would be as productive, if not more productive. And also benefits, well, it’s a positive, all around. It’s a win-win situation, isn’t it? That, you know, employees might feel less stressed.” (P2, female, manager)*

However, participants described ‘engrossing’ work tasks with the added pressure of public scrutiny as a barrier to breaking up sitting time.

*“I think it’s because the work’s more intense here. And you have to concentrate a lot more because you are producing factual figures, you know, for the public to see. So, it is really important that you actually get everything right. So, I think that’s why we tend not to move, ‘cos you get engrossed in a piece of work.” (P6, female, non-manager)*

Across departments, police staff talked about urgent work tasks that have an impact on worker health priorities, whether these be custody cases with a 24-h turnaround, freedom of information request deadlines, emergency calls, or real-time intelligence decision-making.

*“Sometimes we have jobs on where we have people in custody, and we, as you know, you get like the twenty-four hours. So everyone’s trying to work, gather evidence, and it all gets pretty crazy. It’s like, yeah, fast paced.” (P1, female, non-manager)*

*“For police environments full stop, whether it’s CID [Criminal Investigation Department] or whether it’s in here or in the child abuse team, it’s ninety percent of the time… Well, I suppose if we had the will power to listen to the thing [a computer prompt], we'd be alright doing it. But there’s always going to be that one time out of ten, when we’re in the middle of an urgent case with a child at risk, and it [the prompt] comes up, and we’re gonna remove it.” (P5, male, non-manager)*

*“Freedom of information requests, they have a deadline ‘cos they’ve got to get back.” (P2, female, manager)*

#### Perceived autonomy for sitting reduction [reflective and automatic motivation]

Participants perceived that by virtue of them being trusted to manage their own workload, they were likewise trusted to manage their own breaks from sitting and were optimistic about it.

*“Because of our role, we’re very much kind of self-managed.” (P8, female, manager)*

*“I think that if it was what I wanted to do, then I’d just do it whatever, and I wouldn’t worry so much.” (P4, female, manager)*

However, participants were worried that they would be perceived as shirking their work duties.

*“I don’t want people to think that I'm taking the mickey and having an extra break that they're not having.” (P6, female, non-manager)*

Participants were unsure about whether breaking up and/or reducing their sitting time would be acceptable to management.

*“It’s assuring the workforce that the managers are ok with this.” (P2, female, manager)*

Participants generally perceived healthy sitting behaviours to be a responsibility shared jointly by the individual and the organisation, with slightly more onus on the individual.

*“There is a slight onus potentially on peoples’ supervisors or colleagues to go, ‘Do you know you haven’t moved for four hours?’ ((laughs))” (P8, female, manager)*

*“I think ultimately you are responsible for yourself. Because, for example, if I hadn’t told anyone about my back problem, I can’t blame the organisation for my chair not helping. Because I didn’t mention it.” (P7, female, manager)*

*“They’ve [the Force] not necessarily said, ‘Oh, you have got to sit at your desk for seven and a half hours a day, and you cannot move.’ And even if when it’s your lunchtime, ‘You can’t come downstairs.’ Do you know what I mean? ((laughs)). I mean that that, for me, again, it’s a choice. For that half an hour, I tend to sit at my desk and eat my lunch and look at my phone and that’s my choice to do that. I could get up and go for a walk, but I don’t ((laughs)).” (P6, female, non-manager)*

Participants were motivated to change posture due to musculoskeletal discomfort.

*“More people might choose to stand if they suddenly feel like they’re slouching or their back hurts.” (P1, female, non-manager)*

In some cases, older participants believed the health consequences of excessive sitting to be more salient, more so than younger colleagues; yet sometimes, it was the other way around.

*“I think yeah, awareness at an early age. But then again everyone thinks, ‘Oh well. Oh, it won’t happen to me. I'm only in me twenties. I don’t need a pension. I don’t need exercise. I don’t need to watch the way I sit or what I eat.’ You know?” (P2, female, manager)*

*“I don’t think anyone’s overly bothered by it, maybe. And it’s… a lot of them are older as well, and they’ve sort of just done that all of their lives.” (P1, female, non-manager)*

Individuals who had served longer with the Force were perceived as not having any intentions to work differently.

*“[My colleague], who I sit opposite, she tends not to move at all. Like, very rarely. […] And there’s another guy in the office who doesn’t really move either. So I just, so I think that’s… They’ve just got used to being like… ‘Cos they’ve been there for so long.” (P6, female, non-manager)*

## Discussion

The current study expands understanding of the capabilities, opportunities and motivations (i.e., determinants) for sedentary behavior in non-operational police staff. Seven key themes regarding influences on breaking up and reducing sitting time at work in non-operational police staff were identified: 1)*‘Work tasks are seated’* [Physical opportunity], 2)*‘Social norm is to sit’* [Social opportunity], 3) *‘Belief in ability to regulate behaviour’* [Reflective and automatic motivation], 4) *‘Knowledge of health risks’* [Psychological capability], 5) *‘Organisational support’* [Physical and social opportunity], 6) *‘Impact on productivity’* [Reflective and automatic motivation], and 7) *‘Perceived autonomy for sitting reduction’* [Reflective and automatic motivation]*.* The results thus indicate that knowledge of health risks and behavioural regulation (Capability), norms around sitting and organisational support (Opportunity), and perceived outcomes and autonomy (Motivation) are key influences for consideration in an intervention to reduce prolonged sitting in police staff.

Overall, the office culture of police staff was seen as complicit with prolonged sitting. Interpersonal correlates of sedentary time, such as social factors (e.g., norms, cohesion, sense of community), are known to influence sedentary time [15]. Workplace culture specifically has been shown to influence sitting behaviour via the dynamic interplay between an organisation’s values, underlying assumptions, and behaviour [35]. In the present study, managers and employees alike felt they could not be seen to reduce their sitting as they would be perceived as shirking their work. A similar ‘culture of expectation’ equating sitting at one’s desk with work completion has been documented among software engineers [13]. Changing intentions to sit less at work in police staff may thus require an intervention that addresses strongly held beliefs about the social consequences of sitting behaviour change. Uniquely challenging to police staff were urgent job tasks tackling the prevention and solving of crime, saving lives, and providing timely public information. A whole systems approach [36] is needed to address the visible (e.g., behaviour, policy) and not-so-visible (e.g., beliefs about consequences) influences that act to reinforce sedentary police staff work culture [35]. One solution is for employees to work together towards a shared purpose around sedentary behaviour change goals. Incorporating promising behaviour change techniques like social comparison (i.e., team-based competition) may improve behavioural and cardiometabolic outcomes [37]. The inclusion of a team-based intervention with shared goals around sitting reduction is thus recommended in police staff.

Sitting was often performed out of habit by participants, which is consistent with previous studies [33, 38]. One explanation for this is that sitting is perceived as a lower-level action, having more in common with actions like ‘typing’ than a higher-order action like ‘completing work tasks’ [39]. Habits are classed as an individual-level behavioural influence on sedentary time [15]. The process of establishing new sitting habits, therefore, first requires bringing the behaviour into active consciousness [39]. Participants in the current study also described being regularly ‘engrossed in a piece of work’, which made sitting an automatic default behaviour, where they habitually sat and forgot about or were unaware of the length of time spent sitting. Strategies that consciously exploit naturally occurring opportunities to substitute new behaviours for old (e.g., pacing instead of sitting during phone calls) can successfully change norms and reduce sitting time in sedentary office workers as part of multi-component interventions [40, 41]. An intervention to bring prolonged sitting into conscious awareness (e.g., prompts/cues), substitute new behaviours for old (e.g., standing and/or light physical activity for sitting), and reinforce new habits (using rewards and/or incentives), should thus be evaluated in police staff.

Past interventions have often relied on sit-stand desks to assist with substituting sitting habits with standing [23, 40, 41]. Sit-stand desks may be perceived as a ‘key driver’ of reductions in sitting, but decisions to sit or stand may be largely dependent on the task being performed and/or the situational context [42]. In the present study, participants described seated work tasks, sluggish software, and security protocols as reinforcing sitting. However, a lack of confidence in organisational buy-in along with cost were cited as barriers to purchasing sit-stand desks. Public spending scrutiny in the police may factor strongly in the prioritisation of health and wellbeing initiatives. This is a finding that is distinct from studies among other occupational groups [33, 38]. It is clear for police staff that the indoor working environment, considered an environmental-level influence on behaviour [15], is inextricably linked to social influences and wider political factors. Low cost alternatives that make use of the already available opportunities for increased movement and reduced sitting may be more feasible for public sector organisations [43]. For example, prompts/cues are often used in conjunction with active workstations, but can also be effective on their own for reducing and breaking up prolonged sitting [44–46]. Natural breaks away from the desk for drinks or the restroom were recognised by participants in the current study as facilitating periodic activity. An intervention in police staff should thus look to initially incorporate lower cost solutions, such as free or low cost smartphone applications to self-monitor behaviour [47, 48], wearables to prompt standing [49], or computer software to prompt breaks [45], which could later be combined with additional tools and equipment if and when budgets allow. BeUpStanding is a free evidence-informed web-based programme that provides a toolkit to office work teams to deliver an intervention aimed at reducing sitting and promoting occupational health and could be considered in this context [43].

Compounding the issue of organisational buy-in was a lack of knowledge about the cardiometabolic risks that come with prolonged sitting. Knowledge of health problems associated with excess sedentary behaviour was limited mostly to musculoskeletal health and weight gain. Police staff were also not clear on workplace sitting guidelines. Previous evidence suggests this may be why device-measured behaviour in office workers does not reflect sedentary behaviour recommendations to limit sitting bouts to no more than 20–30 min [50]. Providing information about the cardiometabolic health risks of prolonged sitting along with expert guidelines for limiting sedentary behaviour could therefore be included in interventions with police staff.

Finally, participants were conflicted about their own responsibilities for health while at work. Managers admitted setting the example of sitting through lunch breaks at the desk, for example. Despite this, participants held generally positive beliefs about their capabilities to reduce sitting time, but like other office workers, suffered from a lack of intention [7, 33]. On an individual level, participants felt they had few legitimate reasons to move away from the desk apart from tea and bathroom breaks. To address this, participants felt that group-based, organisation-led efforts to reduce sitting would facilitate behaviour change. Previous research has shown that employees may actually prefer if sitting-related changes were organisation-led [51, 52]. Two related studies intervened this way with Australian emergency management staff by installing break prompting computer software that locked employees out of their screens for two minutes every hour while directing them to do short bursts of standing or light intensity physical activity [45, 46]. However, changes in sitting time were not reported and it is thus unclear if this strategy of directed action planning would be effective for sedentary behaviour change. There is a need to evaluate if an organisation-led intervention would bolster intentions to reduce sitting time in police staff.

### Strengths and limitations

A strength of the current study is the mapping of key themes to the COM-B model and TDF framework to deduce theoretical determinants of sedentary behaviour in this understudied occupational group of police staff. Taken forward, this work serves as an evidence base for a theory-driven sedentary behaviour change intervention in police staff. Furthermore, perspectives were gained from both managers and non-managers, as well as a range of departmental roles. However, a limitation of this study was that it only recruited participants from a single police force. Police forces in other regions of England or in other parts of the world may perceive different (or experience to a different degree) influences on workplace sitting reduction. Intentionally, this study was limited to police staff and may not be generalisable to other policing roles. There are many roles in policing that potentially experience high levels of sitting due to travel, paperwork completion, interviews, site visits, and/or court attendance. Similar research needs to be conducted in these occupational subgroups (e.g., police officers) to understand their perceived influences on reducing sitting time at work.

## Conclusions

Overall, police staff have similar influences as other desk-based workers but experience nuanced challenges such as public spending scrutiny, physical and digital security protocols, and the completion of vitally urgent work tasks. This research extends understanding of what influences sitting reduction at work in police staff, as well as contributes to the wider evidence base for sedentary behaviour determinants [36]. A lack of knowledge of sedentary health risks, poor sedentary behaviour regulation capabilities, the normalisation of sedentary working, positive opportunities for organisational support, a mix of concern and optimism regarding productivity outcomes, and uncertainty over perceived autonomy for sitting reduction at work were perceived as important factors by police staff. Environmental changes were perceived favourably, but with minimal confidence as to feasibility due to cost and public accountability. Based on the findings of this study, an intervention to break up and reduce prolonged workplace sitting time in police staff should be organisation-led, raise self-awareness of sitting time, and prompt breaks from prolonged sitting to establish new habits. Working towards shared (team-based) sitting goals and providing education about the health risks of sitting too much, should also be included as part of a multi-component intervention. For police staff, a low cost, theory-driven, multi-component intervention addressing the influences identified here should be developed and evaluated in this unique occupational sub-group.

## Supplementary Information

**Additional file 1.** COnsolidated Criteria for REporting Qualitative studies (COREQ) checklist [18].

**Additional file 2.** Schedule of questions for semi-structured interviews with police staff on influences on breaking up and/or reducing sedentary time at work.

## Data Availability

The qualitative datasets generated and/or analysed during the current study are not publicly available because they contain information that could compromise participant privacy and/or consent. However, they are available from the corresponding author on reasonable request.
