# Supplementary material for: Perceived influences on reducing prolonged sitting in police staff: a qualitative investigation using the Theoretical Domains Framework and COM-B model
Source: BMC Public Health. 2021 Nov 19;21:2126. doi: 10.1186/s12889-021-12019-6 (PMC8605563; doi:10.1186/s12889-021-12019-6)
Supplement: Supplementary file 2 — Additional file 2. Schedule of questions for semi-structured interviews with police staff on influences on breaking up and/or reducing sedentary time at work. [file 12889_2021_12019_MOESM2_ESM.docx]

Additional File 2. Schedule of questions for semi-structured interviews with police staff on influences on breaking up and/or reducing sedentary time at work

| Topic | Questions |
| --- | --- |
| **Welcome script** | Thanks for your time in agreeing to speak with me today. I am interested in hearing your views on prolonged sitting at work. Your answers will help us to design a future workplace intervention.  The interview will be recorded, and will take about 25mins to an hour. I may use quotes from our conversation but they will be kept anonymous and I will provide only a pseudonym as a descriptor. I was hoping to take a few notes as we go along – is that ok?  Please know that you are free to stop at any time should you wish to discontinue the interview. I hope you will find it interesting. Are you happy for us to proceed? |
| **Introductory questions**:  Job Role & background | Tell me about your job role and day-to-day tasks.  *Prompt: Describe the layout of the office and your desk within it.* |
| **Introductory questions:** Sedentary time | How many hours a day do you spend seated? |
|  | When you are seated, how often is it for longer than 20mins at a time?  *Prompt: Never, Some of the time, Most of the time, Always?* |
|  | When you do get up from sitting, how long do you spend away from your desk standing or moving?  *Prompt: Breaks? Non-break movements?* |
|  | Overall, how satisfied are you with the proportion of time you spend sitting, standing and moving at work? |
|  | What is the proportion of responsibility for your health?  *Prompt: Is it 50/50 individual vs employee…?* |
| **Prior Knowledge** | What is your understanding of workplace sedentary behaviour? How would you describe this to others? What words would you use? (Grunseit et al., 2013) |
|  | There are health benefits to being less sedentary (mental physical, and physiological), what do you think about that?  *Prompts:*  *Energy levels*  *Mood*  *Fatigue/Sleepiness*  *Alertness/concentration*  *Energy expenditure*  *Heart health*  *Blood sugar regulation*  *Fat metabolism*  *Musculoskeletal health* |
|  | You may have heard about the health risks associated with too much sitting, what do you think about that?  *Prompt: what health risks have you heard about?*  *Prompt: Have you always felt this way?*  *Prompt: how has your perception changed over time?*  *Prompt: what is significant about this to you?* |
| **Barriers** | What are some of the major barriers for you to reduce sitting at work?  (link to scenarios brought up from earlier questioning)  *Prompts:*  *Time of day*  *Meetings*  *Workflow*  *Task-specific*  *Long hours*  *Work Culture*  *Habit*  *Communications*  *Collaboration*  *Air conditioning*  *Furniture/Layout* |
| **Facilitators** | What would make it possible or easier to sit less or break up sitting time at work?  *Prompts:*  *Environment*  *Individual*  *Organisational*  *Policy* |
| **Prior Knowledge; Barriers & Facilitators:**  Strategies | Tell me about any strategies you know of to reduce sitting time at work.  *Prompt: What are other people in the office doing?*  *Prompt: What do other companies do?*  *E.g.:*  *Standing meetings*  *Talk in person rather than email*  *Central bins/printers*  *Breakroom use/water cooler*  *Use toilets further away*  *Office exercises*  *Incentives/challenges/contests* |
|  | Which of these strategies would be feasible in your workplace? |
|  | What would make these changes sustainable in the long term? |
|  | What might get in the way? |
| **Barriers & Facilitators:**  Workplace culture | On a scale of 1-10, what level of priority does your work place on reducing sitting time?  *Prompt: why have you chosen a ___ and not a 1?*  *Prompt: why have you chosen a ___ and not a 10?* |
|  | Overall, how satisfied are you with this level of priority? |
| **Productivity:**  Impacts of sitting less | If you were able and supported to balance your sitting, standing and moving time at work, what impact (if any) would it have... on your productivity? |
|  | … Job satisfaction? |
|  | … Value as an employee? |
| **Barriers & Facilitators:**  Workplace culture | If sitting less and standing/moving more were made the priority at work, what do you imagine that would look like?  *Prompt: Office energy? What are people doing? Meetings? Competitions?*  *Prompt: How would you feel at first?*  *Prompt: How long would it take you to adjust?* |
| **Impacts:** Non-occupational sitting time | If work was less sedentary, what impact would there be on your current leisure time activities before/after work? |
| **Intervention experience:** Monitoring | How do you monitor your sitting, standing or moving time at the moment?  At work, specifically? |
|  | How would you feel about wearing a very accurate activity monitor for 7 days on your upper leg to measure your sitting, standing and moving at work? |
|  | Any other thoughts about activity monitors? |
| **Closing questions:**  Anything else | Is there anything else you would like to discuss? |
| **Debrief script** | I appreciate your time today in taking part this interview on sitting in the workplace. Once my study is complete the results will be available – you may email me for the details. Also, feel free to get in contact if you have any further questions. Thanks again for your time. |

| Perceived influences on reducing prolonged sitting in police staff: A qualitative investigation using the Theoretical Domains Framework and COM-B model |
| --- |
| *BMC Public Health* |
| Marsha L. Brierley, Brunel University London |
| Lindsey R. Smith, University of Bedfordshire |
| Daniel P. Bailey, Brunel University London |
| Sofie A. Every, Brunel University London |
| Taylor A. Staines, University of Bedfordshire |
| Angel M. Chater, University of Bedfordshire |
| Corresponding author: Marsha Brierley, Brunel University London, marsha.brierley@brunel.ac.uk |
